# Gabapentin for refractory pruritus in severe cutaneous adverse reactions

**DOI:** 10.1016/j.jdcr.2025.01.026

**Published:** 2025-02-21

**Authors:** Claudia Rodriguez Artze, Lily Kaufman, Kalyn Hoffman, Abraham M. Korman

**Affiliations:** a Indiana University School of Medicine, Indianapolis, Indiana; b The Ohio State University College of Medicine, Columbus, Ohio; c Department of Dermatology, The Ohio State University Wexner Medical Center, Columbus, Ohio

**Keywords:** acute generalized exanthematous pustulosis (AGEP), chronic pruritus, drug reaction with eosinophilia and systemic symptoms (DRESS), gabapentin, pruritus, refractory pruritus, severe cutaneous adverse reactions (SCARs), Stevens-Johnson syndrome/toxic epidermal necrolysis (SJS/TEN), treatment

## Introduction

Severe cutaneous adverse reactions (SCARs) include drug reaction with eosinophilia and systemic symptoms, acute generalized exanthematous pustulosis, and Stevens-Johnson syndrome/toxic epidermal necrolysis. SCARs have many known potential complications, including mucocutaneous, ocular, and gastrointestinal. In terms of mucocutaneous complications, severe pruritus may emerge as a chronic complaint that exerts a negative impact on the patient’s quality of life. While gabapentinoids have shown effectiveness in managing pruritus,^1^ clear guidelines for managing chronic pruritus associated with SCAR are lacking, primarily due to the predominant focus of existing research on acute symptomatic treatment. Our series highlights 3 SCAR patients whose pruritus resolved with gabapentin. Therefore, we propose that gabapentin can be used for the treatment of SCAR-associated chronic pruritus.

## Case series

A retrospective analysis identified 3 patients with SCARs from the Ohio State Wexner Medical Center's inpatient dermatology database. These patients presented with posthospitalization pruritus and received treatment with gabapentin between April and December 2023. Data collected included the dosage administered and the clinical outcomes observed (Table I).

**Table I:** Patient characteristics, gabapentin dose, and clinical outcomes

Age	Sex	SCAR	Trigger	Gabapentin dose and timing	Outcome and follow-up
32	Female	AGEP	Amoxicillin	300 mg 3 times daily Initiated during hospital admission 3 d after AGEP presentation	Gabapentin well tolerated without side effects Discontinued after 10 d of treatment Complete resolution prior to follow-up at 6 wk, no recurrence at follow-up 14 mo after discontinuation
47	Female	SJS	Lupus	900 mg daily	Complete resolution within 3 mo
26	Female	AGEP	Clindamycin	900 mg daily	Complete resolution in 1 wk

*AGEP*, Acute generalized exanthematous pustulosis; *SCAR*, severe cutaneous adverse reaction; *SJS*, Stevens-Johnson syndrome.

## Discussion

Research indicates that persistent inflammatory processes can continue after the resolution of SCARs. Drug-tissue complexes trigger sustained inflammation by activating immune cells, including cytotoxic T cells and natural killer cells, leading to ongoing keratinocyte apoptosis and prolonged release of proinflammatory cytokines.^2^^,^^3^ Although visible symptoms may subside, these underlying immunological processes can contribute to lingering effects, including chronic pruritus and skin sensitivity. This ongoing proinflammatory state can further compromise skin barrier function and nerve sensitization, creating a positive feedback loop of itching and scratching. This cycle induces keratinocytes to release thymic stromal lymphopoietin, further perpetuating pruritus. Additionally, abnormal neural innervation in the epidermis, potentially exacerbated by inflammation and massive exfoliation, may also contribute to this sensitization and itching.^4^

We successfully used gabapentin at modest doses to induce full remission in 3 patients with pruritus secondary to SCARs. All patients were treated with gabapentin for only 1 to 12 weeks after which it was tapered without recurrence of pruritus. Gabapentin is a second-generation anticonvulsant that inhibits calcium-mediated neurotransmitter release of excitatory molecules like glutamate, norepinephrine, and substance P.^5^ It also decreases central neural hypersensitization,^4^ exerts anti-inflammatory actions, and influences the affective component of pain.^5^ Considering its multifaceted mechanisms that address the underlying issues faced by patients following SCAR resolution, we propose that this is why it proves effective. While gabapentinoids have demonstrated efficacy in pruritus management,^1^ no data are available on their use for pruritus secondary to SCARs. The successful utilization of gabapentin in alleviating pruritus in 3 patients with SCARs suggests a safe and effective treatment option. However, it is worth noting that gabapentin is renally cleared; thus, when employed for the management of SCARs with potential renal involvement (eg, drug reaction with eosinophilia and systemic symptoms), dermatologists are advised to assess baseline renal function. In cases involving high dosage regimens, periodic reevaluation of kidney function throughout the treatment course should be considered. A limitation of this case series is that it is possible that the pruritus may have been self-limited without further intervention, but this warrants further study.

## Declaration of generative AI and AI-assisted technologies in the writing process

During the preparation of this work, the author(s) used ChatGPT in order to improve language and readability. After using this tool/service, the author(s) reviewed and edited the content as needed and take(s) full responsibility for the content of the publication.

## Conflicts of interest

None disclosed.
